# Fine root decomposition and nutrient release of different age *Caragana intermedia* plantation in alpine sandy land

**DOI:** 10.3389/fpls.2025.1630639

**Published:** 2025-07-30

**Authors:** Qingxue Li, Zhiqing Jia, Lingxianzi He, Xuebin Zhao, Xiuben Yang

**Affiliations:** ^1^ Institute of Ecological Conservation and Restoration, Chinese Academy of Forestry, Beijing, China; ^2^ Qinghai Gonghe Desert Ecosystem Research Station, Qinghai, China; ^3^ Research Institute of Forestry, Chinese Academy of Forestry, Beijing, China

**Keywords:** alpine sandy land, *Caragana intermedia* plantation, fine root, decomposition, nutrient release

## Abstract

A better understanding of fine root decomposition and nutrient release characteristics is essential for accurate assessment and prediction of nutrient cycling in plantation ecosystems. Decomposition bag method was used to study the fine root (1 mm< D ≤ 2 mm, 0.5 mm< D ≤ 1 mm and D ≤ 0.5 mm) decomposition and nutrient release of *Caragana intermedia* plantation with different age (4-, 9-, 11-, 16- and 22-years old) in Gonghe Basin of the Tibetan Plateau. The results showed that (1) The C and K contents of fine root with 1 mm< D ≤ 2 mm and 0.5 mm< D ≤ 1 mm were higher than those D ≤ 0.5 mm. The N content of all fine root was significantly increased (*P*< 0.05), while the K content was significantly decreased (*P*< 0.05) with plantation age. (2) In the first decomposition period (81d), the fine root mass decomposition and nutrient release rate reached more than 50% (except P), and the K release was the fastest (76.86-94.73%). (3) The decomposition coefficient and nutrient release rate increased with fine root diameter. Only the fine root with 1 mm< D ≤ 2 mm decomposition rate decreased (except 9-year-old) with plantation age. (4) The mass loss and nutrient release of fine root were positively correlated with their K, C, N and P contents (*P*< 0.01). RDA analysis showed that the K content of fine root had the highest explanation for the mass loss and nutrient release (68.50-91.50%), followed by the C content (5.10-20.20%), and both reached a very significant level (*P<* 0.01).

## 1 Introduction

Plant roots are important organs that absorb nutrients and water from the soil. They provide convenient conditions for the exchange of matter and energy between plants and soil, and become an important carbon sink and nutrient pool in terrestrial ecosystems (Freschet et al., 2021). In addition, plants complete the ecological process of material circulation and energy flow between the underground part and the soil through root turnover (growth, death, decomposition, and re-growth) (Gill and Jackson, 2000). As a rule, rapid root turnover indicates strong nutrient utilization ability (Luo et al., 2021). This process consumes a large amount of photosynthetic products and releases nutrients, which is an important channel for carbon cycling and soil fertility maintenance (Zhang et al., 2020). Fine root, usually defined as root with a diameter ≤ 2 mm, provide essential functions including nutrient and water acquisition for plants (McCormack et al., 2017). Moreover, fine root has short life cycle, fast turnover, strong metabolism and fast decomposition rate, accounting for the main part of dead roots, and are a major contributor to ecosystem carbon sequestration (Guo et al., 2004). The contribution of fine root to soil carbon pool may be greater than that of above-ground litter (Majdi et al., 2008). Previous studies have shown that about 30-80% of soil organic carbon content is provided through rapid turnover and decomposition of fine root (Ruess et al., 2003). Fine root production, mortality, and turnover patterns are important for the whole plant mediating the carbon allocation strategy (McCormack et al., 2015). Fine root decomposition process transport nutrient between soil and plants, which is the main and stable source for soil matter (such as soil organic carbon) and nutrient elements accumulation (Liebmann et al., 2020; Xu et al., 2022). Therefore, root decomposition is important for nutrient and carbon cycling in forest ecosystem, and it is necessary for soil carbon input, but also a very important role in the self-fertilization of forests (Fu et al., 2021).

Root decomposition is influenced by both climatic and edaphic factors including precipitation, soil temperature and moisture (Santonja et al., 2019). Previous studies have found that root decomposition rate is positively correlated with temperature and precipitation (Zhang et al., 2008), and root decomposition rate in cold and dry areas is the slowest while in the tropics is the fastest (Gargaglione et al., 2019). Root decomposition is also influenced by root diameter and chemical composition, including initial root C and N content (Guo et al., 2008; Sun et al., 2013). The interaction effect among biological factors and environmental also have an impact on fine root decomposition (Liu et al., 2017). In addition, some studies have shown that most studies considered all fine root with diameter less than 2 mm has the same structure and physiological function, which ignores the heterogeneity of the internal structure and function (Guo et al., 2004; Yang et al., 2019). Different branching levels of fine root with different physiological processes, which affect the histochemical composition (Goebel et al., 2011), and the differences in chemical composition between different root branch orders may affect their decomposition rate (Wang et al., 2022). The findings of studies on the fine root decomposition influencing factors are inconsistent, and the underlying mechanisms is unclear, therefore, more experiments are needed, which is essential for understanding nutrient cycling in arid and semi-arid ecosystems.

The alpine region of the Qinghai-Tibet Plateau is the sensitive area of climate change in Asia, and also the ecological security barrier of the whole country and even Southeast Asia. The Gonghe Basin is located in the northeast of the Qinghai-Tibet Plateau, which is a typical representative of desertification in Qinghai Province, and also an important part of the construction of the national “Three-North Protection Forest System”. The ecological environment of this region is fragile, cold, drought and windy are the main characteristics. Vegetation restoration measures have been carried out on sandy land in the Gonghe Basin since 1958 and have obtained good results. *Caragana intermedia* Kuang et H. C. Fu is the main species for vegetation restoration on moving sand dunes in this region. At present, *C. intermedia* plantations with different recovery time are widely distributed on sand dunes. Study the characteristics of fine root decomposition and nutrient release of *C. intermedia* and analyze their relationship with environmental factors, to provide scientific basis for accurate assessment and prediction of nutrient cycle in alpine sandy ecosystem, and provides reference for sustainable management of *C. intermedia* plantation. Therefore, we conducted experiments in different age of *C. intermedia* plantations (4-, 9-, 11-, 16- and 22-years old) to answer the following scientific questions: (1) Whether the fine root decomposition and nutrient release change with the plantations age? (2) Does the fine root diameter affect its decomposition and nutrient release? (3) What are the main influencing factors of fine root decomposition and nutrient release?

## 2 Materials and methods

### 2.1 Study site

This experiment was performed at the Desertification Combating Experimental Site of the Qinghai Gonghe Desert Ecosystem Research Station (99°45′–100°30′E, 36°03′–36°40′N), which altitude is 2871 m. The climate belongs to the transition zone of alpine arid desert and semiarid grassland. The mean annual air temperature is 2.4°C. The mean annual precipitation is 246.3 mm, and the mean annual potential evaporation is 1716.7 mm. The mean annual frost-free period is 91 days, and the total solar radiation is 6631.69 MJ·m^-2^·a^-1^. The mean annual number of windy days is 50.6 days, and the maximum wind speed reaches 40 m·s^-1^. The main vegetation type of the study area is sand-fixing plantations, dominated by the tree species *Populus cathayana* Rehd and *Populus simonii* Carr. and the shrub species *C. intermedia*, *C. korshinskii* Kom., *Salix cheilophila* Schneid., *Salix psammophila* C. Wang et Chang Y. Yang and *Hippophae rhamnoides* Linn. The zonal soil is chestnut soil, and the azonal soils are aeolian sandy soil, meadow soil and bog soil. The soil of *C. intermedia* plantations in this study is aeolian sandy soil (Li et al., 2023).

### 2.2 Experimental design

The field experiment was conducted from June 15, 2022 to August 17, 2024. From June 15 to 20, 2022, fine root samples of *C. intermedia* were dug in 4-, 9-, 11-, 16- and 22-years old plantations (The afforestation methods and initial densities of these plantations are the same). To minimize damage to the shrub, the sampling depth was 0–20 cm. Soil samples were collected simultaneously, with 3 replicates for each plantation age. In the laboratory, the fine root with a diameter greater than 2 mm and the inelastic dead root were removed, and then the fine root samples were washed and divided into 3 diameters, 1 mm< D ≤ 2 mm, 0.5 mm< D ≤ 1 mm and D ≤ 0.5 mm. All fine root samples were dried in the oven at 65°C for 48 h. A part of the fine root was used for the initial nutrient element determination, and the other fine root was cut into small root segments of 5 cm. Then, the 2.000 g fine root sample was weighed with a thousandth balance and putted into a 10×15 cm nylon decomposition bags (80-µm mesh, prevents root growth in the litter bags), marked and sealed. On June 28, 2022, fine root decomposition bags of different plantation ages were buried in the corresponding plantations at a depth of 0–20 cm. From August to April of the following year, the temperature was very low, which resulting the experiment could not be carried out during this period, therefore, fine root samples were collected during the growing season from May to October. In the first year of this experiment, after the fine roots were buried, the soil environment was disturbed, which requires some time to stabilize. Therefore, sampling was conducted only once at the end of the growing season in the first year, and it was defined as the first decomposition stage. During the growing season of the second year (2023, May to October), sampling was conducted approximately every two months, which are the second, third and fourth decomposition periods respectively. In the third year, sampling was conducted only once in the middle of the growing season, which was the fifth decomposition period. Samples were collected on September 17, 2022 (81 days), June 30, 2023 (367 days), August 26, 2023 (424 days), October 22, 2023 (481 days), and August 17, 2024 (780 days). On the sampling day, 15 decomposition bags were collected for each diameter of fine root, and soil samples were collected at the same time, with 3 replicates each plantation age. The samples were taken to the laboratory, the live root in the decomposition bag and the soil attached to the surface of the bag were removed, then the samples were washed under running water and dried in the oven at 65°C for 48h to obtain dry mass. In this study, a total of 1350 bags of fine root samples and 90 soil samples were obtained. The weighed fine root samples were ground and passed through 100-mesh sieve for the determination of C, N, P and K contents. Soil samples were also ground and passed through 100-mesh screens for the determination of soil organic carbon (SOC), soil total nitrogen (STN), soil total phosphorus (STP) and soil total potassium (STK) content.

The soil moisture and soil temperature in 0–20 cm depth of different plantation ages were monitored continuously by ECH_2_O soil moisture monitoring system. As environmental factors, mean monthly soil moisture and soil temperature during the growing season from May to October were analyzed.

### 2.3 Chemical analysis

Fine root C and N content was quantified using a CHNOS Elemental Analyzer (Elemental Analyzer, Elementar Analysensysteme GmbH, Germany). Fine root P and K contents were quantified using an Inductive Coupled Plasma Emission Spectrometer (ICAP 6300 ICP-OES Spectrometer, Thermo Scientific, USA). SOC was determined by potassium dichromate and sulfuric acid method, STN was determined by Semimicro-Kjeldahl Method, STP and STK were obtained with the HF-HCLO_4_-HNO_3_ digestion method using a 6300 ICP-AES (Forestry industry standard of the People’s Republic of China, 1999).

### 2.4 Calculations

Fine root dry mass remaining and nutrients remaining were expressed as follows (Fu et al., 2021):


$$M=\left(M_{t}/M_{0}\right)\times 100\%$$

$$N=\left(N_{t}M_{t}/N_{0}M_{0}\right)\times 100\%$$

Where *M* is mass remaining (%), *M*_0_ is the initial dry weight of fine root, *M*_t_ is the dry weight of fine root after decomposition during time t; *N* is nutrient element remaining (%), *N*_0_ is the nutrient’s initial concentration, and *N*_t_ is the nutrient’s concentration after time t.

The fine root decomposition rate constant k was calculated using the following exponential equation (Olson, 1963):


$$y=M_{t}/M_{0}=ae^{-kt}$$

t is decomposition time (year), and *a* is a fitting parameter.

Time needed to reach 50% and 95% mass loss were estimated using the k value:


$$T_{50\%}=ln_{0.5}/\left(-k\right)$$

$$T_{95\%}=ln_{0.05}/\left(-k\right)$$

### 2.5 Statistical analysis

One-way ANOVA and Duncan’s multiple range test was used to analyze the difference of initial nutrients content and mass remaining of fine root at different plantation ages and diameters. Correlation analysis was used to analyze the relationship among fine root mass loss, nutrient release, fine root nutrient content and soil characteristics. The main influencing factors of fine root decomposition and nutrient release were analyzed by redundancy analysis (RDA). All statistical analyses were conducted using Excel 2019, SPSS 19.0, CANOCO 5.0 and Origin software.

## 3 Results

### 3.1 Initial nutrient content of fine root

The C content of 1 mm< D ≤ 2 mm and 0.5 mm< D ≤ 1 mm fine root was significantly higher than that of D ≤ 0.5 mm fine root (*P*< 0.05) (except 11-year-old). The K content of 1 mm< D ≤ 2 mm and 0.5 mm< D ≤ 1 mm in 11-, 16- and 22-year-old plantations was significantly higher than that of D ≤ 0.5 mm fine root (*P<* 0.05). There was no significant difference in N content of fine root among different diameters (*P* > 0.05) (except 16-year-old). With the plantation age increased, the N content of 3 diameters fine root was significantly increased (*P*< 0.05), and the K content was significantly decreased (*P*< 0.05), the C and P content did not change regularly with the increase of plantation age (**Table 1**).

**Table 1:** Initial nutrients content of different diameter fine root.

Plantation age (Years)	Fine root diameter	C (%)	N (%)	P (g/kg)	K (g/kg)
4	1 mm< D ≤ 2 mm	44.27 ± 0.02Bb	2.49 ± 0.08Aa	0.97 ± 0.02Bb	9.83 ± 0.11Ca
	0.5 mm < D ≤ 1mm	44.32 ± 0.08Db	2.63 ± 0.10Aa	0.96 ± 0.01Ab	9.50 ± 0.25Ca
	D ≤ 0.5 mm	40.51 ± 0.29ABa	2.44 ± 0.01Aa	0.86 ± 0.01Aa	9.18 ± 0.15Ba
9	1 mm < D ≤ 2 mm	44.56 ± 0.21Bb	2.75 ± 0.08Ba	1.18 ± 0.02Ca	8.85 ± 0.65ABa
	0.5 mm < D ≤ 1 mm	44.60 ± 0.07Eb	2.70 ± 0.03Aa	1.11 ± 0.01Ba	8.78 ± 0.02ABa
	D ≤ 0.5 mm	42.30 ± 0.21Ba	2.71 ± 0.01Ba	1.08 ± 0.10Aa	7.86 ± 0.13Aa
11	1 mm < D ≤ 2 mm	43.26 ± 0.26Aa	2.87 ± 0.03Ba	1.14 ± 0.01Cb	9.49 ± 0.05BCc
	0.5 mm < D ≤ 1 mm	43.03 ± 0.11Aa	3.02 ± 0.11Ba	0.94 ± 0.04Aa	9.09 ± 0.19BCb
	D ≤ 0.5 mm	42.31 ± 0.35Ba	2.78 ± 0.01Ba	0.98 ± 0.01Aa	7.22 ± 0.04Aa
16	1 mm < D ≤ 2 mm	44.36 ± 0.03Bb	2.89 ± 0.10Ba	0.86 ± 0.03Aa	8.66 ± 0.20ABb
	0.5 mm < D ≤ 1 mm	43.96 ± 0.11Cb	3.39 ± 0.03Cc	0.92 ± 0.02Aab	9.05 ± 0.05BCb
	D ≤ 0.5 mm	39.20 ± 0.38Aa	3.14 ± 0.02Cb	0.98 ± 0.01Ab	7.19 ± 0.04Aa
22	1 mm < D ≤ 2 mm	43.05 ± 0.32Ab	3.28 ± 0.10Ca	1.11 ± 0.03Cb	9.06 ± 0.10ABCc
	0.5mm < D ≤ 1 mm	43.48 ± 0.04Bb	3.33 ± 0.09Ca	0.92 ± 0.04Aa	8.35 ± 0.11Ab
	D ≤ 0.5 mm	40.76 ± 0.24ABa	3.29 ± 0.02Ca	0.99 ± 0.01Aa	7.20 ± 0.03Aa

Different uppercase letters following values indicate a significant difference among plantation ages; different lowercase letters following values indicate a significant difference among fine root diameters, according to Duncan’s multiple range test (P < 0.05), The same below.

### 3.2 Fine root decomposition

**Figure 1:**
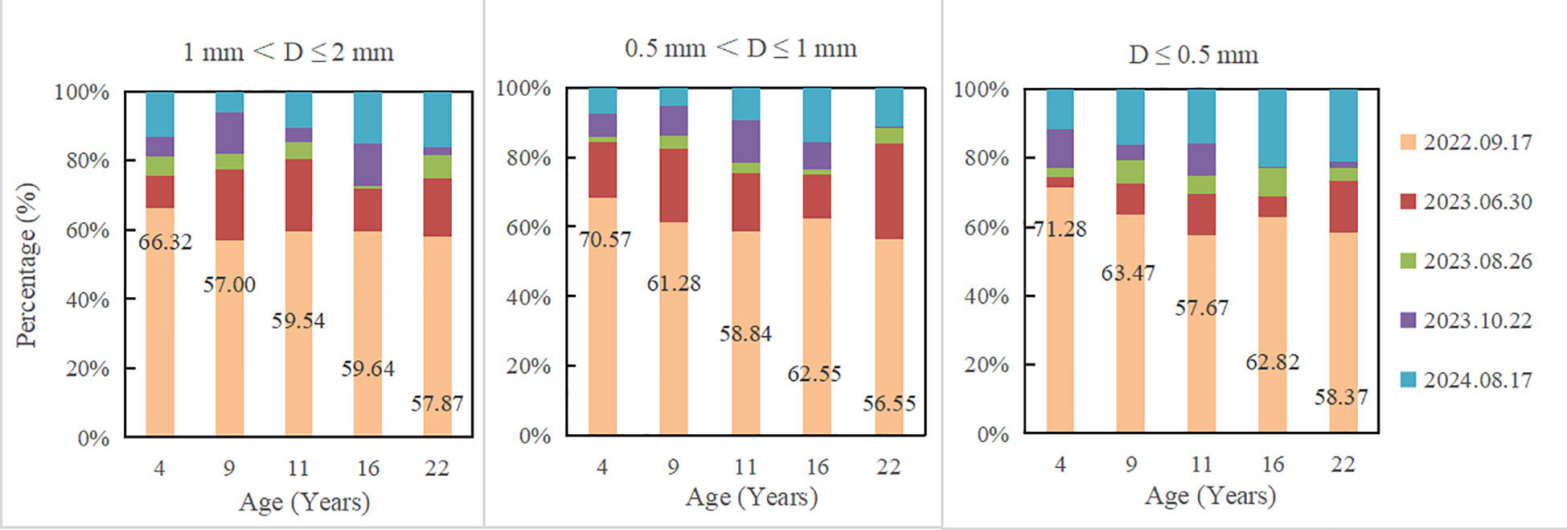
The proportion of fine root decomposition at different sampling date.

Fine root mass remaining decreased with the increase of decomposition time, and the decomposition rate in the first decomposition period (81 days) accounted for the highest percentage in the whole decomposition period, ranging from 56.55 to 71.28% (**Table 2**; **Figure 1**). Fine root mass remaining in all plantations increased significantly with the decrease of fine root diameter (*P<* 0.05). After 780 days of decomposition, fine root mass remaining of 1 mm< D ≤ 2 mm, 0.5 mm< D ≤ 1 mm and D ≤ 0.5 mm were 27.31-36.17%, 28.12-42.36% and 43.24-54.54%, respectively. With the plantation age increased, fine root mass remaining of 1 mm< D ≤ 2 mm increased significantly (*P<* 0.05) (except 9-year-old), and the fine root mass remaining of 0.5 mm< D ≤ 1 mm and D ≤ 0.5 mm changed irregularly.

**Table 2:** Fine root mass remaining in different age *Caragana intermedia* plantations.

Plantation age (year)	Root diameter	Mass remaining of different sampling date (%)				
		2022.09.17	2023.06.30	2023.08.26	2023.10.22	2024.08.17
4	1 mm< D ≤ 2 mm	51.79 ± 0.98Aa	45.09 ± 0.94Aa	40.99 ± 1.17Aa	36.88 ± 1.31Aa	27.31 ± 1.04Aa
	0.5 mm < D ≤ 1mm	54.30 ± 0.61Aa	43.82 ± 0.71Aa	44.81 ± 0.87Ab	40.17 ± 1.13Bb	35.24 ± 0.77Bb
	D ≤ 0.5 mm	62.95 ± 1.12ABb	61.29 ± 0.97Ab	59.99 ± 0.73Ac	54.08 ± 0.73Ac	48.02 ± 0.90BCc
9	1 mm < D ≤ 2 mm	60.65 ± 0.62BCa	46.61 ± 1.04Aa	43.38 ± 0.81Aa	35.12 ± 1.44Aa	30.96 ± 1.45Ba
	0.5 mm < D ≤ 1 mm	61.65 ± 0.97Ca	48.28 ± 1.22Ba	46.17 ± 1.18ABa	40.79 ± 1.90Bb	37.41 ± 1.44Bb
	D ≤ 0.5 mm	71.15 ± 0.97Db	67.01 ± 1.03Bb	63.88 ± 0.98Bb	61.96 ± 1.36Cc	54.54 ± 0.86Cc
11	1 mm < D ≤ 2 mm	59.02 ± 0.64Ba	44.59 ± 0.76Aa	41.25 ± 1.14Aa	38.43 ± 1.83Aa	31.17 ± 0.80 Ba
	0.5 mm < D ≤ 1 mm	57.71 ± 1.64Ba	45.89 ± 0.99ABa	43.75 ± 1.24Aa	34.85 ± 1.39Aa	28.12 ± 1.72Aa
	D ≤ 0.5 mm	67.27 ± 1.27BCb	60.58 ± 0.87Ab	57.56 ± 0.89Ab	52.17 ± 1.58Ab	43.24 ± 1.35Ab
16	1 mm < D ≤ 2 mm	60.47 ± 1.08BCa	52.44 ± 1.12Ba	51.80 ± 0.77Ca	43.78 ± 0.76Ba	33.72 ± 0.52BCa
	0.5 mm < D ≤ 1 mm	60.81 ± 0.94Ca	53.00 ± 0.86Ca	52.17 ± 1.21Ca	47.19 ± 0.98Cb	37.35 ± 0.90Bb
	D ≤ 0.5 mm	65.98 ± 1.31ABb	62.70 ± 1.29Ab	58.18 ± 1.05Ab	58.00 ± 0.82Bc	45.85 ± 0.81ABc
22	1 mm < D ≤ 2 mm	63.06 ± 0.95Ca	52.29 ± 0.79Ba	48.03 ± 0.74Ba	46.52 ± 2.81Ba	36.17 ± 1.25Ca
	0.5mm < D ≤ 1 mm	67.40 ± 0.97Db	51.54 ± 0.82Ca	49.13 ± 1.10BCa	48.92 ± 1.22Ca	42.36 ± 0.90Cb
	D ≤ 0.5 mm	70.01 ± 0.67CDc	62.29 ± 1.30Ab	60.40 ± 1.42Ab	59.33 ± 1.02BCb	48.61 ± 1.13Bc

Different uppercase letters following values indicate a significant difference among plantation ages; different lowercase letters following values indicate a significant difference among fine root diameters, according to Duncan’s multiple range test (P< 0.05), The same below.

The fine root decomposition coefficients of all plantations decreased with the increase of fine root diameter. With the plantation age increased, fine root decomposition coefficients of 1 mm< D ≤ 2 mm decreased slowly (except 9-year-old), and the decomposition coefficient of 0.5 mm< D ≤ 1 mm and D ≤ 0.5 mm first increased and then decreased (**Table 3**). The time of fine root decomposition to achieve 50% and 95% mass loss of 1 mm< D ≤ 2 mm increased with the plantation age increase. The time of fine root decomposition to achieve 50% and 95% mass loss of D ≤ 0.5 mm is much higher than that of the other two diameter fine roots.

**Table 3:** Fine root decomposition coefficient of different stand age predicted by the Olson’s model.

Plantation age (Years)	Root diameter	Model function	R^2^	Decomposition coefficient *k* (year^-1^)	T_50%_ (year)	T_95%_ (year)
4	1 mm < D ≤ 2 mm	y = 59.01e^–0.343t^	0.950^**^	0.343	0.48	7.20
	0.5 mm < D ≤ 1 mm	y = 56.35e^–0.227t^	0.963^**^	0.227	0.53	10.67
	D ≤ 0.5 mm	y = 67.81e^–0.149t^	0.835^*^	0.149	2.04	17.50
9	1mm < D ≤ 2 mm	y = 64.60e^–0.365t^	0.922^**^	0.365	0.70	7.01
	0.5mm < D ≤ 1 mm	y = 63.09e^–0.267t^	0.926^**^	0.267	0.87	9.50
	D ≤ 0.5 mm	y = 74.92e^–0.142t^	0.959^**^	0.142	2.85	19.06
11	1 mm < D ≤ 2 mm	y = 62.18e^–0.337t^	0.985^**^	0.337	0.65	7.48
	0.5 mm < D ≤ 1 mm	y = 64.32e^–0.389t^	0.941^**^	0.389	0.65	6.57
	D ≤ 0.5 mm	y = 73.35e^–0.238t^	0.949^**^	0.238	1.61	11.28
16	1 mm < D ≤ 2 mm	y = 68.52e^–0.313t^	0.924^**^	0.313	1.01	8.36
	0.5 mm < D ≤ 1 mm	y = 66.90e^–0.258t^	0.956^**^	0.258	1.13	10.05
	D ≤ 0.5 mm	y = 72.27e^–0.192t^	0.896^*^	0.192	1.92	13.91
22	1 mm < D ≤ 2 mm	y = 68.18e^–0.293t^	0.993^**^	0.293	1.06	8.92
	0.5 mm < D ≤ 1 mm	y = 67.83e^–0.240t^	0.938^**^	0.240	1.27	10.86
	D ≤ 0.5 mm	y = 74.57e^–0.190t^	0.977^**^	0.190	2.10	14.22

T_50% and_ T_95%_ indicate the predicted time of fine root decomposition to achieve 50% and 95% mass loss, respectively. *P<0.05; **P<0.01.

### 3.3 Fine root nutrients release

**Figure 2:**
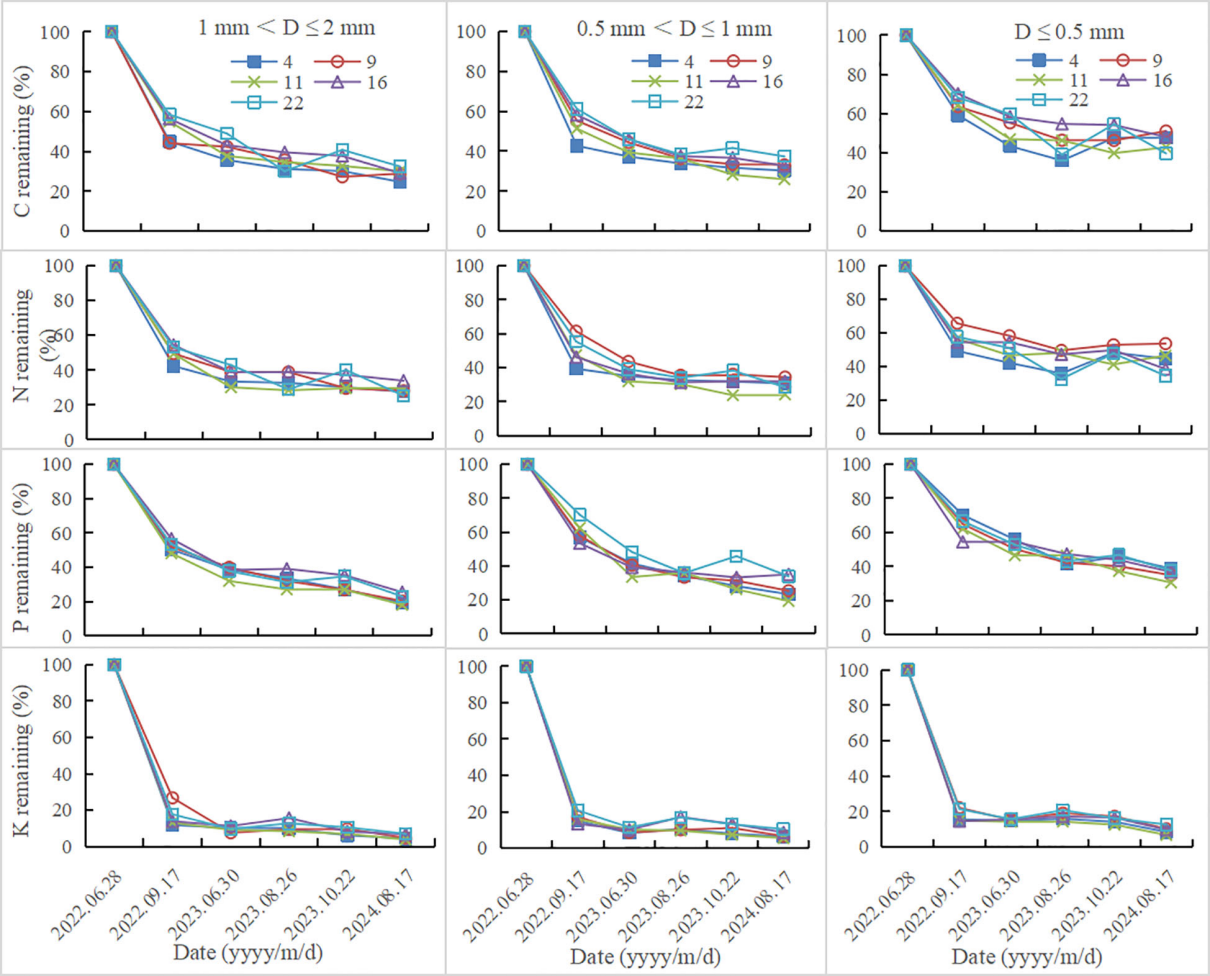
Fine root nutrient remaining at different decomposition stages.

**Figure 3:**
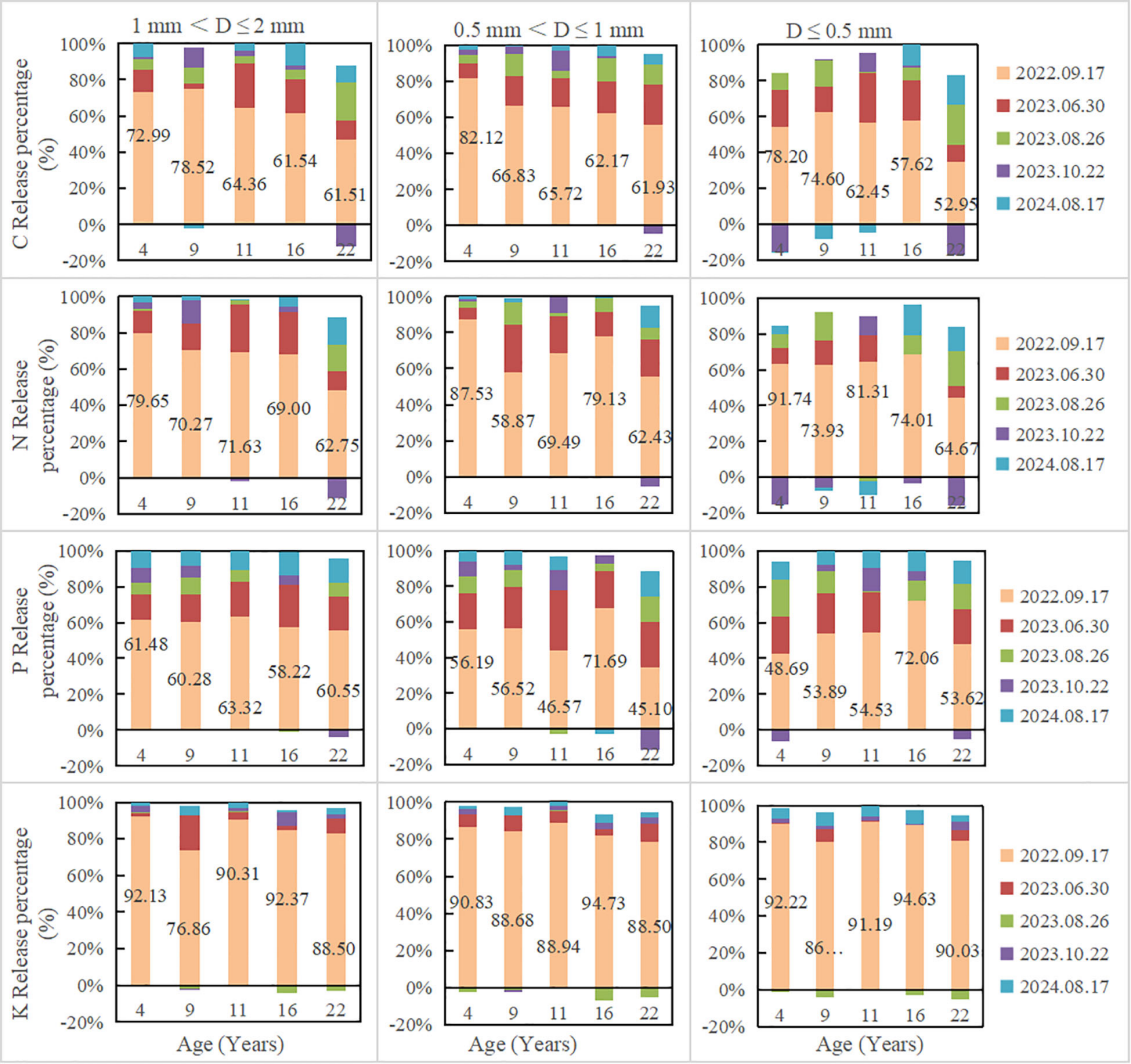
The proportion of fine root nutrients release at different decomposition stages.

Fine root nutrient remaining decreased with the increase of decomposition time. In all plantations, fine root nutrient remaining increased with the decrease of fine root diameter (**Figure 2**). After 780 days of decomposition, fine root N remaining of 1 mm< D ≤ 2 mm, 0.5 mm< D ≤ 1 mm and D ≤ 0.5 mm were 25.25-33.87%, 24.17-34.41%, 34.39-53.52%, respectively; C remaining were 24.70-32.40%, 25.93-37.39%, 39.63-51.06%, respectively; P remaining were 17.96-25.39%, 19.2-34.93%, 30.58-38.78%, respectively; K remaining were 3.87-7.04%, 5.44-10.42%, 8.46-14.63%, respectively. The decomposition rate in the first decomposition period (81 days) accounted for the highest percentage in the whole decomposition period, ranging from 56.55 to 71.28% (**Table 2**; **Figure 1**). In the first decomposition period, the fine root nutrient release rate accounted for the highest percentage in the whole decomposition period. The release rate of K element was the fastest, and the percentage of K release rate in the first decomposition period was 76.86-94.73%, followed by N (58.87-87.53%) and C (52.95-82.12%), P is relatively slowly (45.10-72.06%) (**Figure 3**). The enrichment of C, N and P elements occurred in the late decomposition period, and the enrichment of D ≤ 0.5 mm fine roots in 4 and 22 year old plantations was more obvious. The enrichment of K element began in the middle stage of decomposition.

### 3.4 Relationship between fine root decomposition and environmental factors

**Figure 4:**
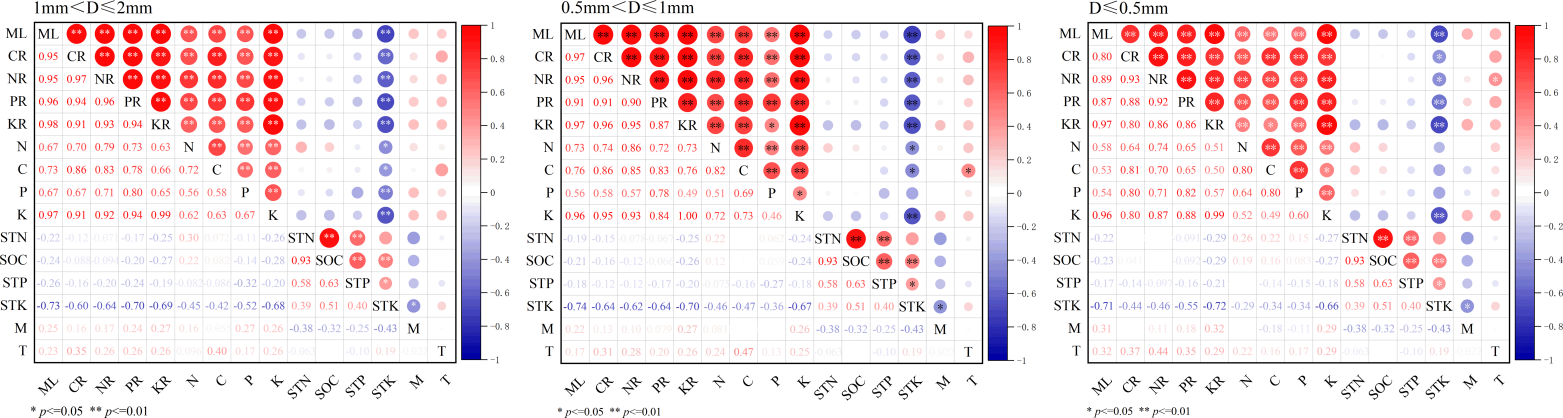
Pearson correlation of fine root mass loss, nutrients release, fine root nutrients content and soil characteristics. ML, mass loss; CR, C release; NR, N release; PR, P release; KR, K release, M, soil moisture, T soil temperature, the same below.

**Figure 5:**
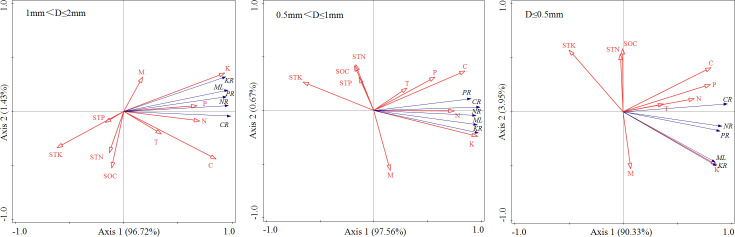
RDA analysis of mass loss, nutrients release and environmental factors of different diameter fine root.

The mass loss and nutrient release of 3 diameter fine roots were positively correlated with their K, C, N and P contents (*P<* 0.01), and negatively correlated with the STK content (*P*< 0.05) (**Figure 4**). The results of RDA analysis of mass loss, nutrient release and environmental factors of different diameter fine root showed that the total interpretation of axis 1 and axis 2 of 1 mm< D ≤ 2 mm, 0.5 mm< D ≤ 1 mm, and D ≤ 0.5 mm fine root were 98.15%, 98.23% and 94.28%, respectively (**Figure 5**). The K content of different diameter fine roots had the highest explanation for the mass loss and nutrient release, reaching 84.60%, 91.50% and 68.50%, respectively, followed by the C content (12.00%, 5.10% and 20.20%), and all reached a very significant level (*P*< 0.01) (**Table 4**).

**Table 4:** Importance ranking and significance test results of environmental variable interpretation.

Fine root diameter (mm)	Impact factors	Explains (%)	Contribution (%)	*F*	*P*
1 mm < D ≤ 2mm	K	84.6	86.2	126	0.002**
	C	12	12.2	77.3	0.002**
	STN	0.8	0.8	6.1	0.032*
	SOC	0.2	0.2	1.2	0.27
	STP	0.2	0.2	1.3	0.254
	P	0.1	0.1	1.1	0.304
	STK	0.1	0.1	1	0.34
	N	0.1	0.2	1.2	0.236
	M	<0.1	<0.1	0.4	0.58
	T	<0.1	<0.1	0.3	0.578
0.5 mm < D ≤ 1 mm	K	91.5	93.1	247	0.002**
	C	5.1	5.2	32.7	0.002**
	N	0.8	0.8	5.9	0.024*
	STP	0.4	0.4	3.5	0.084
	T	0.2	0.2	2	0.158
	M	0.1	0.1	1.1	0.302
	STK	0.1	0.1	1.3	0.248
	SOC	<0.1	<0.1	0.5	0.456
	P	<0.1	<0.1	<0.1	0.866
	STN	<0.1	<0.1	<0.1	0.912
D ≤ 0.5 mm	K	68.5	72.6	49.9	0.002**
	C	20.2	21.4	39	0.002**
	STK	1.6	1.7	3.5	0.058
	N	1.9	2.1	5	0.058
	T	0.6	0.6	1.5	0.246
	SOC	0.7	0.8	2.1	0.164
	STN	0.5	0.5	1.4	0.256
	P	0.3	0.3	0.8	0.428
	M	<0.1	<0.1	<0.1	0.848

**P<0.01, *P<0.05.

## 4 Discussion

### 4.1 Initial nutrient content of fine root

In this study, we found that the C and K contents of fine roots with 1mm< D ≤ 2 mm and 0.5mm< D ≤ 1 mm were higher than those D ≤ 0.5 mm, which was consistent with previous studies. For example, Wang et al. (2022) reported that the lower-order fine root (commonly< 0.5 mm in diameter) with lower carbon concentration, while the higher-order roots contained higher concentrations of initial C content (Yang et al., 2019). Jani et al. (2015) reported that the initial carbon content of coarse roots (>1 mm diameter) was significantly higher than fine roots (<1 mm diameter) for *Trifolium incarnatum* and *Vicia villosa* Roth. With the increase of plantation age, the N content of all fine roots was significantly increased (*P*< 0.05), and the K content was significantly decreased (*P*< 0.05). *C. intermedia* is a leguminous shrub, the large number of rhizobia with high nitrogenase activity in its root, which can fix free nitrogen in the air (Li et al., 2014). Potassium involves in the adjustment of membrane penetration in host cell and a series of assimilation process, promotes plant growth and improves photosynthetic efficiency, therefore ensures the nodule formation and nitrogenase activity in legumes (Hu and Schmidhalter, 2005). Previous studies on the allocation and cycling characteristics of main nutrients for *C. intermedia* in this region showed that the N content of root system was significantly negatively correlated with fine root K content, indicating that the nitrogen fixation process of the root system consumes its own K, and mainly consumes the K of the fine root with D ≤ 2 mm (Li et al., 2023). It probably because that with the increase of plantation age, the fine root nitrogen fixation ability increased, and it needs to consume more K element, which leads to a significant increase in the N content and a significant decrease in the K content.

### 4.2 Fine root decomposition and nutrients release

In the first decomposition period (81d), the fine root mass decomposition and nutrient release rate reached more than 50% (except P release), and the K element release was the fastest (76.86-94.73%). It is generally believed that in the early stage of decomposition, the decomposition process is greatly influenced by abiotic actions, and soluble substances such as carbohydrates in fine root leachate rapidly, while in the later stage of decomposition, the decomposition process is mainly influenced by biological action, most soluble compounds are consumed, and insoluble substances such as lignin and cellulose remain, which are slowly degraded by microorganisms (Lv et al., 2020). Zhou et al. (2022) also reported that annual percentage mass loss of root during the first stage contributed about 75%–85% of the total mass loss over the 2-year decomposition period. Liu et al. (2021) found that the fine root of *A. halodendron* decomposed rapidly in the initial period, more than 40% of the mass was lost within the first 33 days. The release rate of P is relatively slowly, evidence has been reported suggesting that P is tended to be immobilized during root decomposition in forests (Gargaglione et al., 2018). Güsewell and Freeman (2005) reported that litter with N/P > 22 tends to be immobilized during decomposition. In this study, the N/P was greater than 22, and N/P increased with the decomposition time, which might be one of the reasons for the slower release of P element.

The K released right from the first decomposition period without immobilization in 3 fine root diameters because K is not the structural component of plant litter and not bound into any known organic compounds (Bhattarai et al., 2022), it is mainly present in the solution of plant cells in the form of ions that are released directly mainly through leaching (Osono and Takeda, 2004). Xu et al. (2006) also observed that the K releases rapidly from the beginning of decomposition.

The decomposition coefficient and nutrient release rate of fine root increased with the increase of fine root diameter, and the decomposition rate of fine root with D ≤ 0.5 mm was the slowest. Previous studies have shown that lower-order roots (commonly< 0.5 mm in diameter) are not lignified and are rich in nutrients, especially for root tips, which have higher levels of acid-insoluble compounds, while higher-order roots are the opposite. These difficult to decompose acid-insoluble compounds are the main factor causing the slower decomposition rate of lower-order roots (Sun et al., 2016; Yang et al., 2019). In addition, the smaller the diameter of the lower-order roots, the greater the proportion of root bark tissue, the higher the content of secondary defense substances such as tannin in the root bark cell wall, and the more difficult it is to decompose (Chen et al., 2001). Other studies suggested that the lower-order fine roots are more easily colonized by ectomycorrhizal fungi and the chitin rich mycorrhizal sheath may reduce the fine roots decomposition (Guo et al., 2008). Studies on the root decomposition of various temperate tree species also show that the content of acid-insoluble substances in roots with a diameter of less than 0.5 mm is greater than that in roots with a diameter of 0.5-3.0 mm (Hendricks et al., 2000). Xiong et al. (2013) also indicated that the decomposition rate of low-order root was significantly negatively correlated with the content of acid-insoluble substances, very fine root (< 0.5 mm) decayed slower than 0.5-2.0 mm root in temperate and subtropical tree species. Fan and Guo (2010) reported that lower-order roots (commonly< 0.5 mm in diameter) decomposed at a slower rate than higher-order roots (> 0.5 mm in diameter). The very fine roots (< 0.5 mm) with rapid turnover rate but slow decomposition, may promote the accumulation of soil C and N in terrestrial ecosystems (Fan and Guo, 2010).

### 4.3 The main influencing factors of fine root decomposition

The mass loss and nutrient release of fine root were positively correlated with their K, C, N and P contents (*P*< 0.01). Among the indicators monitored in this study, the K content of fine root had the highest explanation for the mass loss and nutrient release (68.50-91.50%), followed by the C content (5.10-20.20%), and both reached a very significant level (*P<* 0.01). This result was consistent with previous studies, for example, Wang et al. (2017) reported that fine root decomposition rate of *Pinus massoniana* was significantly positively correlated with the initial concentrations of N, P, K and Ca. Wang et al. (2022) reported that the fine root decomposition constant was significantly positively correlated with the initial C concentrations. Yang et al. (2019) also found that the low decomposition rate of the lower-order fine root was due to their relatively low carbon concentration. K, as a non-structural element, is mainly dissolved in plant cells and is a nutrient element that is more prone to leaching in litter (Aponte et al., 2012). K is also crucial for maintaining osmotic potential and activating enzymes. Previous studies have also shown that the release rate of K element from fine roots is faster than that of other elements, because K has a high solubility and can exude rapidly from the decomposed fine roots (Cao et al., 2018). In this study, K and C had a significant impact on the decomposition and nutrient release of fine root, it’s probably because the highly soluble K element and the easily decomposing soluble carbohydrates had a stimulating effect on the microbial community, thereby promoting its decomposition (Fan and Guo, 2010). Soil microbial community is the main decomposer in the soil decomposition system and also affect the decomposition of fine roots (Chapin et al., 2002). However, this study did not measure microbial indicators, and their impact on fine root decomposition needs further investigation. In addition, soil animals feed on fungi and bacteria, break up organic matter, spread microbial propagules and change nutrient availability, which affecting the activities of microbial communities, and then influencing the decomposition process of fine roots (Smith et al., 2009; Ruan et al., 2005). In this study, decomposition bags pore size was 80-µm, and the influence of soil animals on fine root decomposition was ignored. There are still many deficiencies in comprehensively and systematically revealing the process and mechanism of fine root decomposition with different plantation ages.

## 5 Conclusion

In conclusion, the C and K content of large diameter fine root (1mm< D ≤ 2 mm and 0.5mm< D ≤ 1 mm) were significantly higher than those of small diameter fine root (D ≤ 0.5 mm). With the increase of plantation age, the N content of all fine root was significantly increased, while the K content was significantly decreased. Fine root mass decomposition and nutrient release in the early period were the fastest (81d), which exceeded 50% of the whole decomposition period (except P release), and the K release was the fastest, reaching 76.86-94.73%. The decomposition coefficient and nutrient release rate of fine root increased with the increase of fine root diameter, and the time of mass loss to achieve 50% and 95% of fine root with D ≤ 0.5 mm is much higher than that of the other two larger diameter fine roots. The effect of plantation age on fine root decomposition was not significant, only the fine root with 1 mm< D ≤ 2 mm decomposition decreased with the increase of plantation age (except 9-year-old). In this study, the fine roots decomposition and nutrient release were positively correlated with their K, C, N and P contents, and the K content had the greatest effect on fine root decomposition and nutrient release, followed by the C content. Our findings provide a solid theoretical foundation and data support for the accurate assessment of nutrient cycling of *C. intermedia* plantation in alpine sandy land.

## Data Availability

The original contributions presented in the study are included in the article/**Supplementary Material**. Further inquiries can be directed to the corresponding author/s.
